# Hypoxia and Selective Autophagy in Cancer Development and Therapy

**DOI:** 10.3389/fcell.2018.00104

**Published:** 2018-09-10

**Authors:** Ioanna Daskalaki, Ilias Gkikas, Nektarios Tavernarakis

**Affiliations:** ^1^Institute of Molecular Biology and Biotechnology, Foundation for Research and Technology-Hellas, Heraklion, Greece; ^2^Department of Biology, University of Crete, Heraklion, Greece; ^3^Department of Basic Sciences, Medical School, University of Crete, Heraklion, Greece

**Keywords:** autophagy, cancer, ERphagy, HIFs, hypoxia, mitophagy, mTOR, pexophagy

## Abstract

Low oxygen availability, a condition known as hypoxia, is a common feature of various pathologies including stroke, ischemic heart disease, and cancer. Hypoxia adaptation requires coordination of intricate pathways and mechanisms such as hypoxia-inducible factors (HIFs), the unfolded protein response (UPR), mTOR, and autophagy. Recently, great effort has been invested toward elucidating the interplay between hypoxia-induced autophagy and cancer cell metabolism. Although novel types of selective autophagy have been identified, including mitophagy, pexophagy, lipophagy, ERphagy and nucleophagy among others, their potential interface with hypoxia response mechanisms remains poorly understood. Autophagy activation facilitates the removal of damaged cellular compartments and recycles components, thus promoting cell survival. Importantly, tumor cells rely on autophagy to support self-proliferation and metastasis; characteristics related to poor disease prognosis. Therefore, a deeper understanding of the molecular crosstalk between hypoxia response mechanisms and autophagy could provide important insights with relevance to cancer and hypoxia-related pathologies. Here, we survey recent findings implicating selective autophagy in hypoxic responses, and discuss emerging links between these pathways and cancer pathophysiology.

## Introduction

Maintenance of oxygen homeostasis is essential for cellular and organismal survival. Insufficient oxygen availability or hypoxia, represents a common feature of several pathologic as well as physiologic processes. While naturally occurring hypoxia is indispensable for the early onset of mammalian embryonic development, it also contributes to the pathogenesis of several diseases such as stroke, heart failure, and cancer. In any case, evolutionary conserved cellular and systemic responses to oxygen limitation have been developed in organisms as diverse as the nematode *C. elegans* and humans. Such responses attempt to restore tissue oxygenation through sustaining the vascular system and increasing cardiac output. To cope with oxygen deprivation, cells respond by adjusting their metabolic and bioenergetic demands through a number of oxygen-sensing pathways including the hypoxia-inducible factors (HIFs) family of transcription factors -dependent and -independent responses. HIFs belong to the basic helix-loop-helix/PER-ARNT-SIM (bHLH/PAS) family of proteins, which form specific heterodimeric complexes between the HIFα and HIFβ subunits. Specifically, HIF-1α, HIF-2α, and HIF-3α isoforms comprise an oxygen-sensitive alpha subunit which is heterodimerized with the constitutively expressed beta subunit of HIF-1β (Majmundar et al., 2010; Schito and Semenza, 2016). While most of the hypoxia-responsive genes rely on HIF-1α and HIF-2α heterodimerization with HIF-1β in the nucleus, little is known about HIF-3α regulation and function upon hypoxia. Structural differences as well as tissue-specific expression indicate functional discrepancies between the three isoforms (Koh et al., 2011; Masson and Ratcliffe, 2014; Soni and Padwad, 2017). When oxygen is abundant HIF-1α is rapidly targeted for proteasomal degradation. During this process, prolyl hydroxylases (PHDs) catalyze the hydroxylation of conserved prolyl residues in HIF-1α promoting its ubiquitination through von Hippel Lindau (pVHL) protein and ultimately its degradation from the proteasome. Low oxygen levels inhibit PHDs activity allowing stabilization and nuclear translocation of HIF-1α which is subsequently heterodimerized with HIF-1β (Eales et al., 2016). Hereafter, heterodimerized HIF-1α with HIF-1β will be referred as HIF-1.

Notably, HIF-1-dependent responses are extensively studied, whereas a role of HIF-1-independent responses to hypoxia has just emerged. Particularly, unfolded protein response (UPR) and the mechanistic or mammalian target of rapamycin (mTOR) can act in parallel with, or even substitute HIF-1 activity (Wouters and Koritzinsky, 2008). Based on the severity and duration of hypoxia, each HIF-1- and non-HIF-1-mediated response can involve multiple alternative pathways such as apoptosis and autophagy among others, to promote hypoxia resistance. A balancing act of such responses heavily relies on the coordinated regulation of autophagy by HIF-1, UPR, and mTOR in response to hypoxia (Fang et al., 2015). Deregulation of both mTORC1 and mTORC2 complexes of mTOR signaling represents a common feature of various human solid tumors (Kim et al., 2017). Growing body of evidence shows that inhibition of mTOR protein kinase results in autophagy activation which in turn can be either beneficial or detrimental for tumor survival (Brugarolas et al., 2004; Levy et al., 2017; Paquette et al., 2018; Singh et al., 2018). Similarly, autophagy can be stimulated by UPR induction in response to endoplasmic reticulum (ER) stress and hypoxia (Senft and Ronai, 2015). Equivalently, it appears that HIF-1 possesses diverse regulatory roles in autophagy activation (Mazure and Pouyssegur, 2010). Interestingly rather than being regulated by HIF-1, autophagy *per se*, can regulate HIF-1 stability (DePavia et al., 2016). This reciprocal regulation of autophagy and HIF-1 activity can account for opposing roles of autophagy activation in various human tumors.

Depending on the type of stimulus and cellular damage, mTOR, UPR, and HIF-1 constitute protective responses converging on autophagy. While the molecular mechanism underlying autophagy process has been extensively reviewed elsewhere, little it is known about the role of HIF-1, UPR, and mTOR in hypoxia-induced autophagy (Kaur and Debnath, 2015; Farré and Subramani, 2016; Dikic and Elazar, 2018). Compelling evidence suggests that autophagic degradation of cellular components is triggered in response to hypoxic stress. Coordination of cellular energy releasing and consuming processes such as mitochondrial oxidative phosphorylation (OXPHOS), glycolysis and protein synthesis upon hypoxia has been assigned to autophagy (Mazure and Pouyssegur, 2010; Eales et al., 2016). Toward this direction, proteins, lipids and whole organelles are targeted for degradation, not only to replenish cell with new “building material” but also to readjust cellular function. Specifically, organelles such as mitochondria, peroxisomes and endoplasmic reticulum (ER) among others, are highly targeted by selective autophagy processes named mitophagy, pexophagy, and ERphagy/reticulophagy, respectively. Interestingly, selective degradation of such organelles can be specifically and differentially regulated upon hypoxia when compared to induction of the same processes by other stresses. The existence of specialized mechanisms for selective autophagy induction upon hypoxia highlights the significance of such mechanisms for hypoxic adaptation. Latest findings related to the aforementioned types of selective autophagy triggered upon hypoxia/HIF-1 induction in mammalian systems, is going to be the focus of this manuscript.

## Hypoxia response mechanisms converge on autophagy

**Figure 1:**
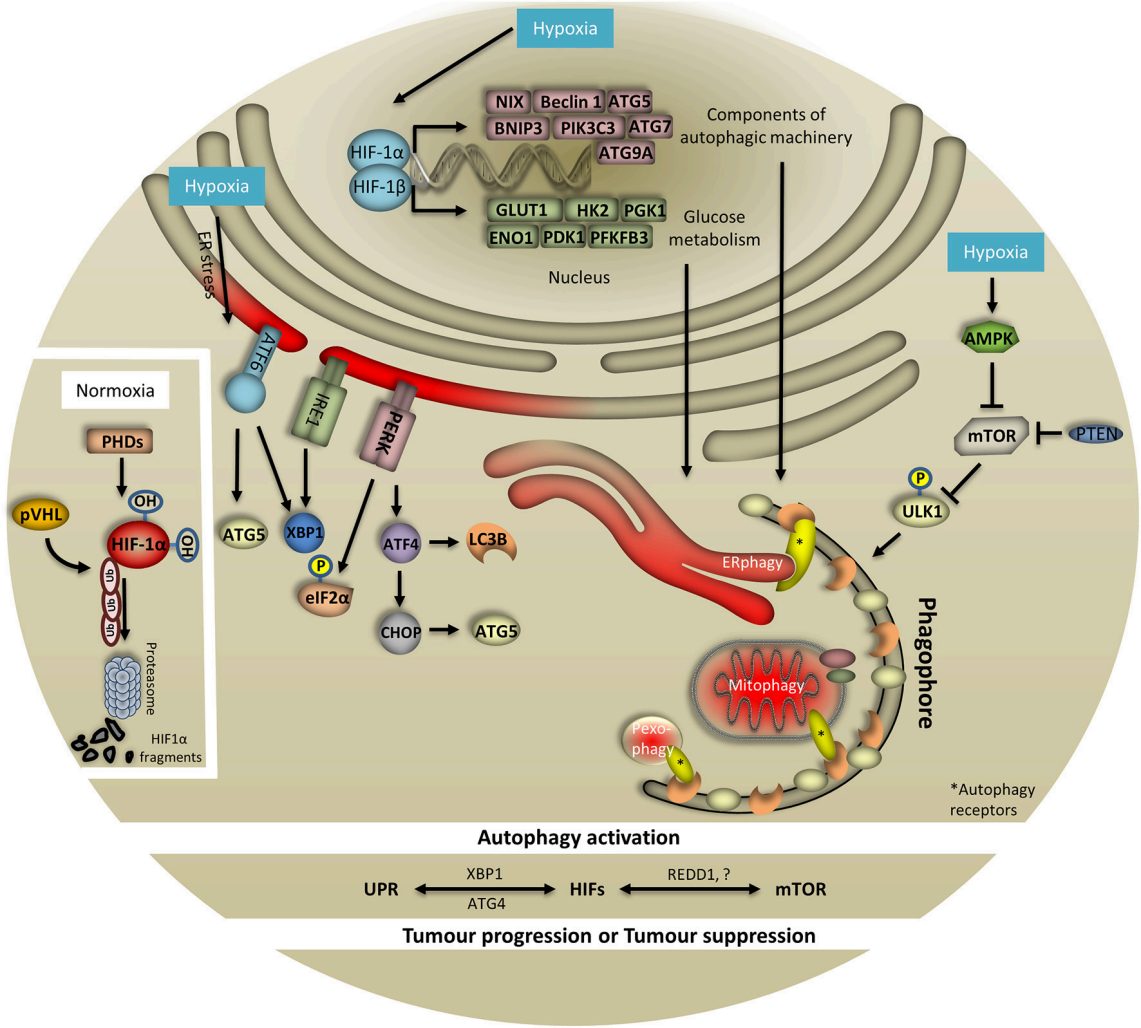
HIF-1, UPR, and mTOR coordinate hypoxia-induced autophagy. To cope with low oxygen levels, cells evoke different oxygen-sensing pathways such as HIF-1, UPR, and mTOR which are tightly coordinated and regularly converge on autophagy. When oxygen is abundant HIF-1 is hydroxylated by prolyls hydroxylases (PHDs) and ubiquitinated by von Hippel Lindau (pVHL) protein. These combined actions result in HIF-1 degradation from the proteasome. In response to hypoxia, HIF-1 is stabilized and translocates to the nucleus to initiate the transcription of multiple genes involved in autophagy, glucose metabolism and mitochondria respiration, among others. Importantly, HIF-1 regulates essential genes for the assembly and function of the autophagy machinery. Particularly, expression of NIX, Beclin 1, ATG5, BNIP3, PIK3C3, ATG7, and ATG9A has been documented to be HIF-1-dependent. Notably, the expression of key glycolytic enzymes that are direct targets of HIF-1 has also been associated with the autophagy process. Specifically, expression of GLUT1, HK2, PGK1, ENO1, PDK1, and PFKFB3 has been reported to be HIF-1-dependent. Surprisingly, in response to oxygen and/or glucose deprivation, expression of HIF-1 targets is linked with the autophagy process. In this respect, hypoxia-induced AMPK negatively regulates mTOR signaling which in turn drives autophagy initiation through ULK1 phosphorylation (bulk and selective autophagy i.e., ERphagy, mitophagy, pexophagy shown here). Similarly, PTEN can inhibit mTOR upon hypoxia and subsequently control autophagy. To this direction, during periods of oxygen limitation cells respond by activating UPR which requires the action of three signaling proteins comprising PERK, IRE1, and ATF6. In the course of PERK-mediated response, eIF2α is phosphorylated to prevent mRNA translation. Moreover, upon hypoxia PERK controls regulation of ATF4 and CHOP which in turn orchestrate the activity of autophagy-related proteins. The contribution of IRE1/XBP1 and ATF6 arms of UPR in autophagy upon hypoxia has just emerged. Although the exact associations with and within UPR, mTOR, HIF-1, and autophagy remain elusive upon hypoxia, identification of XBP1, ATG4, and REDD-1 among others, further supports this notion and highlights the complexity of the system.

Due to the multitude of intracellular and environmental stimuli (such as oxidative stress, unfolded proteins, nutrient availability, radiation, heat sock, hypoxia etc.) that an organism has to cope with, it is imperative to maintain the robustness and specificity of cellular protective mechanisms. Among these stimuli, hypoxia and nutrient deprivation represent a common feature of the tumor microenvironment. Adaptation and survival of tumor cells in such a heterogenic microenvironment requires the coordination of several stress response pathways including HIF-1, mTOR, UPR, and autophagy. Of particular importance is the role of hypoxia-induced autophagy in tumor progression. Emerging evidence suggests that various signaling pathways converge on autophagy in response to hypoxia. In this regard, recent progress has demonstrated that autophagy plays an essential role in hypoxic reprogramming of tumor cells conferring resistance to chemotherapy drugs and fostering tumor survival. While hypoxia affects many aspects of tumor biology, the degree to which HIF-1, mTOR, and UPR pathways converge on autophagy to promote survival remains unclear (Figure 1).

### HIF-1 and autophagy

In response to hypoxia, activated HIF-1 regulates the transcription of numerous hypoxia-responsive genes, most of which are implicated in energy and oxygen homeostasis (such as glucose metabolism and oxidative phosphorylation etc.). Conversely, glucose deprivation as well as mitochondrial damage can also activate HIF-1, suggesting its feedback regulation by a set of interrelated signaling events possibly through mTOR, UPR, and autophagy. Despite the complexity of HIF-1 regulation, the role of HIF-1 in tumor progression through autophagy has long been appreciated (Masoud and Li, 2015). Recent data suggest that HIF-1-dependent regulation of both selective and bulk autophagy is mediated by changes in the expression of numerous of its target genes. Importantly, core autophagic machinery components have been shown to lie among HIF-1 targets. To this direction, HIF-1-dependent regulation of BCL2 and adenovirus E1B 19 kDa-interacting protein 3 (BNIP3), BNIP3-like (BNIP3L)/NIX, Beclin 1, Phosphatidylinositol 3 kinase catalytic subunit type 3 (PIK3C3), ATG9A, ATG5, and ATG7 has already been documented (Zhang and Ney, 2009; Azad and Gibson, 2010; Cerrada et al., 2013; Gui et al., 2016; Abdul Rahim et al., 2017; Zhou et al., 2018). Rather than directly targeting autophagic components, HIF-1 can also regulate autophagy by altering glucose metabolism. In this respect, HIF-1 promotes glucose metabolism through the regulation of glucose transporters−1/3 (GLUT1/3), hexokinases (HK1/2), lactate dehydrogenase (LDHA), phosphoglycerate kinase 1 (PGK1), pyruvate dehydrogenase kinase 1(PDK1), enolase 1 (ENO1), and 6-phosphofructo-2-kinase/fructose-2,6-bisphosphatase 3 (PFKFB3) among others, although the contribution of each of them to the autophagy process remains elusive (Schofield and Ratcliffe, 2004; Papandreou et al., 2006; Denko, 2008; Masoud and Li, 2015).

Interestingly, the association with and within HIF-1-related glycolytic enzymes and autophagy has just emerged. During periods of oxygen limitation, autophagy activation controls glucose uptake through GLUT1 activity and its plasma membrane expression (Roy et al., 2017). Recent findings suggest that PGK1 plays a crucial role in autophagy activation through direct binding to VPS34/Beclin1/ATGL14 complex upon glutamate and oxygen deprivation (Qian et al., 2017a). This interaction relies in part, on the protein kinase activity of PGK-1 which phosphorylates Beclin at S30. Compelling evidence indicates that PGK-1 reciprocally regulates glycolysis and autophagy during tumorigenesis (Li X. et al., 2016; Qian et al., 2017b). In line with this, it has been shown that human T cells lacking PFKFB3 redirect their metabolism from glycolysis to the pentose phosphate pathway (PPP) resulting in high NADPH production and low ROS levels which in turn block autophagy (Yang et al., 2013). On the contrary, either genetic or pharmacological inhibition of PFKFB3 constrains the ability of HCT-116 colon adenocarcinoma cells to uptake glucose, accompanied by autophagy induction (Klarer et al., 2014; Shi et al., 2017). Of note, the association of PDK1 with unc-51-like autophagy-activating kinase 1 (ULK1) was shown to regulate autophagy in acute myeloid leukemia (AML) cell lines. Specifically, chemical inhibition of PDK1 with dichloroacetophenone was sufficient to prevent this interaction and subsequently suppress autophagy (Qin et al., 2016). Contrary to the previous study, hypoxia-mediated recruitment of AKT to mitochondria increases PDK1 activity through its phosphorylation on Thr346, which in turn inhibits autophagy in tumor cells (Chae et al., 2016). Culminating effects of multiple factors confound the regulatory role of PFKFB3 and PDK1 in autophagy. Thus, further investigation is required in both a cell-specific and condition-dependent fashion. Furthermore, cancer cells lacking ENO1 enter a catabolic state with increased tricarboxylic acid (TCA), fatty acid oxidation (FAO), and OXPHOS, followed by ROS-induced autophagy (Capello et al., 2016). Recently, it has also been shown that glucose starvation in neonatal rat ventricular myocytes (NRVMs) stimulates autophagy through HK2-mediated inhibition of TORC1 (Roberts et al., 2014). Since autophagy induction by hypoxia and glucose deprivation share common factors including HIF-1 and mTOR, the contribution of each factor to the autophagy process remains enigmatic. Recent findings suggest that mTOR /P70S6K (P70S6-kinase) signaling axis phosphorylates PHD2 at Ser125 and potentiates its activity. On the contrary, PP2A/B55α dephosphorylates PHD2 at Ser125 and reduces its activity. These combined actions control PHD2 enzymatic activity conferring autophagy-mediated hypoxia adaptation of colorectal cancer cells (CRC) in a HIF-1-dependent manner (Di Conza et al., 2017). Collectively, these findings strongly suggest the existence of an unexplored interconnection between mTOR and HIF-1 target genes which impinge on glucose metabolism and in turn control the autophagy process.

### Hypoxia and mTOR regulation of autophagy

The mTOR signaling pathway plays an essential role in maintaining protein synthesis and metabolic homeostasis in response to low energy production as well as hypoxia and nutrient deprivation. As previously mentioned, cells reduce mitochondrial OXPHOS and favor glycolysis to keep pace with energy supply and demand at low oxygen levels. Loss of energy as well as nutrient balance activate AMP-activated protein kinase (AMPK) and negatively regulate mTOR signaling, which in turn results in autophagy induction through ULK1 phosphorylation at Ser317 and Ser777 (Jung et al., 2010; Kim et al., 2011). While hypoxia-induced mTOR inhibition has been largely appreciated, the extent to which mTOR signals to autophagy in response to hypoxia is poorly understood (Vadysirisack and Ellisen, 2012; Fang et al., 2015). Recent findings showed that sustaining cardiac function upon hypoxia/reoxygenation (H/R) injury relies on autophagy and apoptosis inhibition, in part through the Akt/mTOR signaling axis and miR-21 (Huang et al., 2017). Similarly, a cardioprotective role after H/R injury has been proposed for miR-221 which inhibits autophagy through mTOR signaling (Chen Q. et al., 2016). Interestingly, patient with Crohn's disease exhibit diminished inflammatory response and mTOR signaling followed by induction of autophagy in response to hypoxia (Cosin-Roger et al., 2017).

Questionably, a growing body of evidence focuses on hypoxia-mediated regulation of mTOR signaling in several pathological conditions. For instance, in human prostate cancer cells PTEN-deficiency, which leads to a constituvely active mTOR, reduces hypoxia tolerance. Additionally, loss of eukaryotic initiation factor 4E binding proteins 1/2 (4E-BP1/2) enhances tumorigenesis in a prostate cancer mouse model which is accompanied by increased vascularization and reduced number of hypoxic cells. These findings point toward the notion that 4E-BPs can be targeted for efficient tumor therapy of PTEN-deficient cancer cells (Ding M. et al., 2018). Next, lymphocytes exposed to hypoxia dampen lipogenesis and promote lipid oxidation through mTOR signaling (Yin et al., 2017). Notably, hypoxia-induced cellular acidification as a consequence of imposed metabolic adaptation restrains the circadian clock through mTOR inhibition (Walton et al., 2018). In addition, tuberous sclerosis complex 1 and 2 (TSC1/TSC2) and regulated in development and DNA damage response 1 (REDD1) proteins are not required for mTOR inhibition in hepatocytes exposed to hypoxia (Wolff et al., 2011). While it is well accepted that mTOR signaling regulates autophagy, direct evidence showing the contribution of autophagy to such pathologies remains elusive.

### Hypoxia and UPR regulation of autophagy

Apart from HIF-1 and mTOR, autophagy acts as an essential node regularly integrated by UPR in response to ER and hypoxic stress (Bi et al., 2005; Fang et al., 2015). Of note, hypoxic stress prevents the formation of disulphide bonds and suppresses proper protein folding in the ER (Rozpedek et al., 2016). To cope with hypoxia-induced proteotoxicity, cells elicit increased UPR which relies on the action of three established signaling proteins including inositol-requiring protein 1 (IRE1), protein kinase RNA(PKR)-like ER kinase (PERK), and activation transcription factor 6 (ATF6) (Urra et al., 2016). However, the link between UPR and autophagy during periods of limited oxygen availability is poorly understood. In the course of PERK-mediated responses, loss of BiP association with PERK evokes phosphorylation of eukaryotic initiation factor 2α (eIF2α) at Ser51 and subsequently inhibition of mRNA translation (Rozpedek et al., 2016). Importantly, tumor cells lacking eIF2α exhibit increased sensitivity to hypoxia-induced ROS production (Rouschop et al., 2013). Similarly, survival of hypoxic tumor cells has been attributed to autophagy induction through PERK-regulated activation of transcription factor 4 (ATF4) and CCAAT-enhancer-binding protein homologous protein (CHOP). Both ATF4 and CHOP transcription factors control the activity of autophagy-related proteins such as microtubule-associated protein1 light chain 3β (MAP1LC3B/LC3B) and autophagy related gene 5 (ATG5) (Rouschop et al., 2010). Previous studies have shown that hypoxia-induced expression of lysosomal-associated membrane protein 3 (LAMP3) in human tumor cell lines evolves activation of the PERK arm of the UPR (Mujcic et al., 2009). Accordingly, the activity of LAMP3 has been linked with tumor metastasis and poor prognosis independently of HIF-1 (Mujcic et al., 2013; Nagelkerke et al., 2013). In parallel, an autophagy-related cytoprotective role of IRE1 and its downstream target X-box binding protein 1 (XBP1) against hypoxia and tumor growth has only recently emerged (Hetz et al., 2009; Margariti et al., 2013; Chen X. et al., 2014; Fang et al., 2015).

Previous studies have shown that breast cancer cell lines lacking XBP1 exhibit attenuated tumorigenesis due to impaired assembly of XBP1/HIF-1 transcriptional complex and substantial inhibition of downstream hypoxia-responsive genes expression (Chen X. et al., 2014). In addition, it has been reported that co-occupancy of the promoter region of vascular endothelial growth factor A (VEGFA) by ATF4, XBP1, and HIF-1 is indispensable for its expression (Pereira et al., 2014). Given the significance of tumor vascularization for its growth and relapse, it is appealing to further study HIF-1 and UPR co-responsiveness in tumorigenesis. Whether autophagy and XBP1/HIF-1 transcriptional co-occurrence are interrelated with tumorigenesis under the conditions studied, remains to be determined. In this context, interactions between autophagy and ATF6-dependent expression of CHOP and XBP1 have also been documented (Mei et al., 2013). Arguably, the IRE1/XBP1 and ATF6 arms of UPR-induced autophagy are the least studied, therefore further investigation is required to clarify the role of these arms in hypoxia-induced autophagy (Yan et al., 2015).

## Hypoxia-induced selective autophagy

Mitochondrial number, function and overall homeostasis are widely affected by hypoxia. This can be explained by the fact that oxygen deficiency causes a major metabolic switch: OXPHOS dampens and glycolytic pathways are active, in turn. Under aerobic conditions, the main production source of adenosine triphosphate (ATP) is oxidative phosphorylation which is performed by the electron transport chain (ETC) components inside mitochondria. Oppositely, oxygen shortage under hypoxia, renders ETC dysfunctional, thus unable to produce adequate amounts of ATP. Toward this direction, anaerobic glycolysis is prompted to replenish cellular ATP demands. Except for ATP, reactive oxygen species (ROS) are also generated mainly through the ETC. Interestingly it was shown that increased ROS levels produced upon hypoxia play the major role in the signaling cascade that mediates HIF-1 nuclear translocation and stabilization. On the other hand, excessive ROS cause cellular damage and ultimately cell death. To cope with hypoxia-induced mitochondrial damage, cells evoke increased mitophagy rates to keep a healthy mitochondrial pool. Lowering mitochondrial mass upon hypoxic conditions not only protects against excessive ROS production but also tears apart inactive/useless organelles and recycles their constituents, providing necessary building blocks for other cellular processes.

Selective elimination of mitochondria, known as mitophagy, occurs through the activation of various pathways/mechanisms, such as the phosphatase and tensin homolog-induced kinase 1 (PINK1)/PARKIN pathway and the chaperone-, receptor- and lipid-mediated mitophagy (Ploumi et al., 2017). To date, accumulating evidence shows that receptor-mediated mitophagy is the main type of mitophagy activated upon hypoxia. Several proteins participate in this process; however, the components that function as receptors have the most important regulatory role. Therefore identification of specific mitophagy receptors is a crucial step toward understanding the underlying molecular mechanisms. To date, Bnip3-like/NIP3-like protein X (BNIP3L/NIX), Bcl-2/Adenovirus E1B 19 kDa-interacting protein 3 (BNIP3), and FUN14 domain-containing protein 1 (FUNDC1) are the mitophagy receptors reported to be activated under hypoxic conditions in mammals (Sowter et al., 2001; Bellot et al., 2009; Liu et al., 2012).

### FUNDC1-mediated mitophagy

FUNDC1 is expressed in all higher eukaryotes and in almost every tissue. Localization studies revealed that it is an outer mitochondrial membrane (OMM) protein which contains three α-helix transmembrane domains. Its N- terminus is exposed to the cytoplasm whereas the C-terminus lies in the intermembrane space (IMS) of mitochondria (Liu et al., 2012). FUNDC1 is enriched in the mitochondria-associated membrane (MAM) upon hypoxia. Interestingly, small amounts of the protein are also found in the ER-mitochondria contact sites under normoxic conditions. Interestingly, functional-domain analysis revealed an LC3-interacting region (LIR) motif in the cytoplasmic N′ terminus of FUNDC1. This domain mediates the FUNDC1- light chain 3 (LC3) associations in a non-canonical conformation and is indispensable for mitophagy induction upon hypoxia. The function of FUNDC1 as a mitophagy receptor under hypoxia is PINK1/Parkin independent and highly specific. This is evident by the fact that depletion of FUNDC1 did not affect either general autophagy or mitophagy induction upon hypoxia-irrelevant stressors such as starvation (Liu et al., 2012). Detailed mechanistic insight revealed that, under normoxia, FUNDC1 is phosphorylated on its LIR motif by both the proto-oncogene tyrosine-protein kinase Src (Src) and casein kinase 2 (CK2) kinases at Tyr18 and Ser13, respectively. FUNDC1 phosphorylation at these sites and especially at Tyr18 inhibits its association with LC3 (Kuang et al., 2016). These phosphorylation events alter the stereochemical properties of FUNDC1 and decrease its binding affinity for LC3 whereas increase its affinity for binding on other targets (Lv et al., 2017).

On the other hand, upon hypoxia induction, the aforementioned kinases are both dissociated from FUNDC1 through yet not fully understood mechanisms and the levels of phosphorylated FUNDC1 is highly reduced (Chen G. et al., 2014). The inactivation of Src under hypoxic conditions is mediated by a single phosphorylation event, taking place at Tyr416. As a result, phosphorylation on this site blocks FUNDC1 phosphorylation at Tyr18 (Ozkirimli and Post, 2006; Mishra et al., 2009). Importantly, inactivation of both Src and CK2 kinases is mandatory before mitophagy is activated. This inactivation is necessary as: first, only the fully dephosphorylated form of FUNDC1 is the one that binds LC3-II and induces mitophagy and second, the two kinases exhibit functional compensation. Upon hypoxia, FUNDC1 dephosphorylation is promoted by its preferential association with a mitochondrial phosphatase, phosphoglycerate mutase family member 5 (PGAM5). PGAM5 interacts with FUNDC1 and triggers its dephosphorylation at Ser13 as was recently shown in Hela cells (Chen G. et al., 2014). Dephosphorylation of FUNDC1 at this site triggers its association with LC3, followed by mitophagy activation (Wei et al., 2015). PGAM5 and subsequently PGAM5-FUNDC1 associations are multiply regulated. Both in the presence and absence of oxygen, PGAM5 activity is dynamically regulated by Bcl-2-like 1 (BCL2L1/BCL-xL). Under normoxia, BCL2L1/ BCL-xL, which is also an OMM protein, does not physically associate with FUNDC1 but binds PGAM5 through its BH3 domain. This direct binding of BCL2L1/ BCL-xL on PGAM5, renders it inactive, thus unable to dephosphorylate FUNDC1 at Ser13. Furthermore, BCL2L1/BCL-xL by tethering PGAM5 also decreases its availability, thus the interaction of the second with FUNDC1. As a result, FUNDC1-mediated mitophagy is inhibited as evidenced by the decreased association of FUNDC1 with LC3. This function of BCL2L1/ BCL-xL is independent of Beclin 1(BECN1) (Wu H. et al., 2014). Detailed analysis showed that the Bcl-2 homology 3 (BH3) domain of BCL2L1/ BCL-xL is needed but is not sufficient to induce mitophagy. Under hypoxia, on the other hand, BCL2L1/BCL-xL is degraded and PGAM5 is released. Unbound PGAM5 is prone to physically interact with FUNDC1 and trigger mitophagy, as previously described. The involvement of BCL2L1/ BCL-xL in FUNDC1-mediated mitophagy control upon hypoxia is a unique feature of this protein and does not account for every anti-apoptotic component, such as B-cell lymphoma 2 (BCL2) (Chen G. et al., 2014; Wu H. et al., 2014).

*In vitro* analysis has also revealed that phosphorylation of FUNDC1 at Ser17 increases the interaction of the protein with LC3-II by about three-fold (Lv et al., 2017). This phosphorylation is performed by ULK1 which directly interacts with FUNDC1 and is critical for mitophagy induction under hypoxia. Despite the fact that modifications at Ser17 and Tyr18 are very adjacent, still, they oppositely affect mitophagy induction. Thorough analysis of this phenomenon revealed that SRC-mediated phosphorylation is dominant to and suppresses ULK1 phosphorylation at Ser17 when both events are present (Wu W. et al., 2014). All these phosphorylation events in the cytoplasmic region of FUNDC1 highlight the importance of post-translational modifications and relevance to FUNDC1-mediated mitophagy control.

Apart from the post-translational modifications that regulate FUNDC1-mediated mitophagy upon hypoxia, the receptor is additionally regulated at the post-transcriptional and pre-translational level. This type of regulation is mainly under the control of miRNAs and more specifically of miR-137 which is expressed mostly in the brain. miR-137 binds on the 3′ UTR of FUNDC1, thus post-transcriptionally represses its expression. Subsequently, reduced FUNDC1 protein levels lower the number of FUNDC1-LC3 associations and decrease mitophagy rates. Interestingly, this effect was reversed when a FUNDC1 variant, containing a mutation on the miR-137 binding site on its 3′ untranslated region (UTR), was overexpressed. Upon hypoxia, miR-137 expression is decreased compared to normoxia, allowing mitophagy to be induced (Li et al., 2014). Strikingly, a few studies support that the protein levels of FUNDC1 initially drop upon hypoxia induction (Liu et al., 2012). Despite the fact that not much is known about FUNDC1 transcriptional regulation yet, the notion that FUNDC1 is not regulated transcriptionally, in contrast to other mitophagy receptors such as BNIP3/NIX, prevails (Wei et al., 2015; Williams and Ding, 2015). Since FUNDC1 is not transcriptionally regulated upon hypoxia and its protein levels are reduced, it is possible that miR-137 only partially regulates FUNDC1 expression. The controversial findings regarding miR-137 downregulation and FUNDC1 levels drop in the initiation of hypoxia suggest that additional mechanisms regulate FUNDC1 mRNA stability and expression upon hypoxia, other than the miRs.

A recently identified mechanism could explain this paradox. In this respect, FUNDC1 is targeted by a mitochondrial E3 ubiquitin ligase, membrane-associated ring finger (C3HC4) 5 (MARCH5), for ubiquitination and subsequent degradation. As initially perceived, the levels of FUNDC1 quickly declined upon hypoxia and this effect could be reversed upon treatment with either the proteasomal inhibitor, MG132, or an autophagic flux inhibitor, chloroquine (Chen Z. et al., 2017b). Interestingly, at the initial steps of the hypoxic response, MARCH5 homo-oligomers decrease and MARCH5 shifts toward forming associations with FUNDC1, thus degrading it. A deeper understanding of the MARCH5-dependent ubiquitination and targeted degradation of FUNDC1 revealed K119R as the main ubiquitination site on FUNDC1. MARCH5 physically interacts with FUNDC1 through residues that belong to the cytoplasmic compartments of both proteins. This interaction mediates FUNDC1 ubiquitination, as previously described (Chen Z. et al., 2017a). Furthermore, it is shown that MARCH5-dependent degradation of FUNDC1 is independent of Parkin and precedes the dephosphorylation events at Tyr18 which activate FUNDC1. This implies that mitophagy is decreased at the onset of hypoxia, allowing cells to maintain their mitochondrial mass at a quite high level. However, if hypoxia is prolonged or becomes more severe, mitophagy escalates and mitochondrial mass drops. The signaling cascade and the hypoxic duration required to regulate these pathways remain elusive (Chen Z. et al., 2017b).

**Figure 2:**
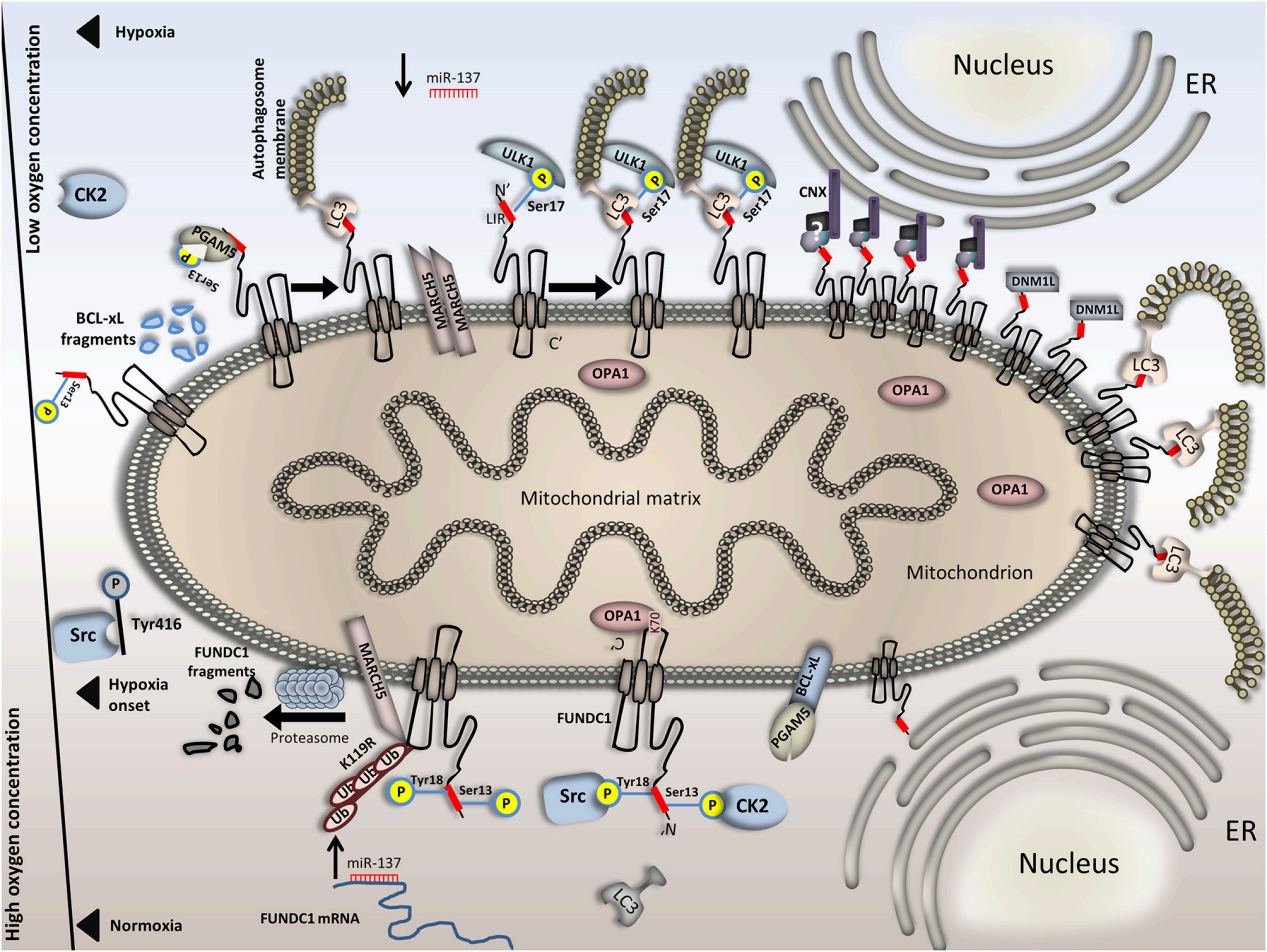
Regulation of FUNDC1-mediated mitophagy in normoxia versus hypoxia. FUNDC1 is inactivated upon normoxia by two phosphorylation events at Tyr18 and Ser13. Phosphorylation at these sites is accomplished by the Src and CK2 kinases, respectively and renders the LIR motif of FUNDC1 inaccessible to LC3. Also, OPA1 binds FUNDC1 in the intermembrane space, promoting mitochondrial fusion. miR-137, which targets the 3′ UTR of FUNDC1 mRNA is upregulated upon normoxia, thus blocking protein synthesis. In parallel, BCL-xL binds PGAM5 and renders it dormant and additionally, the E3 ubiquitin ligase physically interacts with FUNDC1, ubiquitinates it and triggers its proteasomal degradation. This effect is also evident just at the onset of hypoxia. Following, Src kinase is inactivated through phosphorylation on its Tyr416 residue. Src inactivation further blocks Tyr18 phosphorylation on FUNDC1. Moreover, OPA1 dissociates from FUNDC1 and miR-137 is downregulated. FUNDC1 binds PGAM5 phosphatase which cleaves the remaining Ser13 phosphorylation on the first. Interestingly, PGAM5 and CK2 antagonistically bind on FUNDC1. Stoichiometric alterations on FUNDC1 upon hypoxia render its binding affinity for PGAM5 stronger than for CK2. Concomitantly, owing to BCL-xL degradation in response to hypoxia PGAM5 is not trapped on the OMM anymore. Complete dephosphorylation of FUNDC1 triggers LC3 binding and mitophagy onset. Under conditions of prolonged or severe hypoxia, additional mechanisms further increase mitophagy rates. For example, MARCH5 dimerization blocks FUNDC1 degradation and the levels of the second increase. Besides, FUNDC1 phosphorylation at Ser17 by ULK1 increases its affinity for LC3 and last, the CNX-dependent FUNDC1 accumulation in the MAM further boosts mitophagy. This is also facilitated by both DNM1L recruitment and increased affinity of FUNDC1 for LC3 binding. In the bottom half of the Figure, processes that take place under normoxia are illustrated and in the top half, processes under hypoxia. The axis in the left part of the Figure is representative of the available oxygen concentration. Events in the top half (hypoxia) are presented in a specific sequence; when moving from left to right, the severity or duration of the hypoxic event is increased.

Additionally, MARCH5 ubiquitinates proteins such as Dynamin-1-like protein (DNM1L)/ dynamin-related protein 1(Drp1) which participate in mitochondrial fission (Chen Z. et al., 2017b). Mitochondrial fission is a prerequisite for mitophagy events to take place, at least during Pink1/Parkin- mediated mitophagy which is mainly induced upon mitochondrial depolarization (Twig and Shirihai, 2011; Palikaras et al., 2015). Moreover, FUNDC1 has been linked to enhanced mitochondrial recruitment of DNM1L as well as to higher fission rates. This was found to be dependent on both the presence of FUNDC1 in the MAM and the associations it forms with calnexin (CNX). This observation can be explained by data showing that FUNDC1 accumulates in the MAM upon hypoxia and forms indirect associations with the ER protein CNX through its N- terminus. Besides, indications point toward the view that the associations between FUNDC1 and CNX are important for driving the subcellular localization of FUNDC1 on the MAM (Wu et al., 2016a,b). Even though the components that mediate such an association have not been revealed yet, FUNDC1 is not enriched in this region when CNX is absent. Next, if the hypoxic stress persists, FUNDC1 disassociates from CNX and preferentially binds to DNM1L directly, thus triggering mitochondrial fission. Interestingly, not only depletion of either FUNDC1 or DNM1L is detrimental for fission as expected, but also depletion of CNX. Following these events, FUNDC1 binds to LC3 and promotes mitophagy. Although partially understood, this newly identified (CNX-FUNDC1-DNM1L) axis gives a satisfactory understanding of fission and mitophagy coupling upon hypoxia (Wu et al., 2016b). Complementary studies show that FUNDC1 directly interacts with DNM1L through its cytoplasmic end in a LIR-independent fashion. Interestingly, FUNDC1 can also directly associate with the inner mitochondrial membrane and intermembrane space protein optic atrophy 1 (OPA1). OPA1 regulates mitochondrial fusion and was found to interact with FUNDC1 on K70 residue lying in the intermembrane space. The association between OPA1 and FUNDC1 can also regulate mitochondrial fission, as under stress conditions, such as FCCP treatment, FUNDC1-OPA1 association attenuates, in contrast to the FUNDC1-DNM1L association. The phosphorylation status of FUNDC1 can become the decisive point in regulating the balance of the formed associations. The mechanism which regulates whether FUNDC1 associates with either OPA1 or DNM1L and the balance between the two different associations plays an important role in the determination of mitochondrial fusion versus fission upon stress. Despite the fact that direct involvement of OPA1 in FUNDC1-mediated mitophagy upon hypoxia has not been revealed yet, evidence supporting a role of OPA1 in mitochondrial fusion regulation upon hypoxia already exists (Sanderson et al., 2015; Chen M. et al., 2016). Hence, whether mitochondrial fission is a pre-requisite for FUNDC1-mediated mitophagy upon hypoxia or not remains unexplored. Some interesting questions that arise are the following: first, whether MARCH5-dependent degradation of DNM1L acts on the same pathway with FUNDC1- mediated mitophagy or not, and second whether their ubiquitination level is the critical point that regulates mitophagy rates upon hypoxia. Likewise, FUNDC1 phosphorylation on Tyr18 is sufficient to induce mitophagy upon hypoxia even without the presence of mitochondrial fragmentation. This implies that fission events are not required for mitophagy onset upon hypoxia but most probably, enhance the rate of the already ongoing mitophagy events (Kuang et al., 2016). Figure 2 summarizes key information pertinent to the mechanisms described in this section.

### BNIP3/BNIP3L-mediated mitophagy

BCL2 and adenovirus E1B 19kDa-interacting protein 3 (BNIP3) and BNIP3-like (BNIP3L)/NIX are two pro-apoptotic proteins which localize to mitochondria and share many common characteristics and functions. BNIP3 contains one transmembrane domain and localizes on the outer mitochondrial membrane. Its C- terminus is exposed inside mitochondria whereas its N-terminus faces the cytoplasm (Ray et al., 2000). Transcriptionally, expression of both genes is highly elevated upon hypoxia in a HIF-1- dependent manner. Specifically, the HIF-1-dependent transcriptional activation of BNIP3 is further enhanced by Ras and E2F-1, while dampened by nuclear factor kappa-light-chain-enhancer of activated B cells (NF-kB) and retinoblastoma protein (Rb) which act antagonistically to reduce BNIP3 transcription (An et al., 2006; Tracy and MacLeod, 2007; Tracy et al., 2007; Shaw et al., 2008; Yurkova et al., 2008). Moreover, the Forkhead box O3 (FOXO3) and CREB-binding protein (CBP) also participate in the HIF-1-dependent transcriptional control of BNIP3, while both the Tumor protein p53 (p53) and CBP are regulators of the HIF-1-dependent NIX transcriptional control. Interestingly, the exact factors that participate in the transcriptional regulation of BNIP3 may be cell-specific. Indeed, it was recently shown that FOXO3 negatively regulates NIX under hypoxic conditions in a Cbp/p300-interacting transactivator 2 (CITED2)-dependent manner (Guo et al., 2001; Lee et al., 2017). This finding raises doubts as to whether FOXO3 similarly influences BNIP3, in a context-specific manner.

Recently, it has been suggested that both proteins play important roles in the regulation of hypoxia-induced autophagy. Initially, it was shown that depletion of both BNIP3 and BNIP3L totally abrogated hypoxia-induced autophagy in CCL39 cells. This finding coupled with the fact that autophagy induction promotes cell survival upon hypoxia, rendered the two proteins potential pro-survival factors. In addition, both proteins obtain complementary functions. This is evident by the fact that NIX depletion by itself does not severely affect mitophagy induction upon hypoxia. Despite the fact that BNIP3 depletion has a stronger impact on mitophagy, only depletion of both components can totally abrogate hypoxia-induced autophagy (Bellot et al., 2009). Furthermore, BNIP3 and BNIP3L mechanistically trigger autophagy by regulating the Bcl-2-Beclin complex. Specifically, under normoxia the formation of either BCL-xL-Beclin or Bcl-2-Beclin complexes inhibit autophagy. On the other hand, under hypoxia, these complexes dissociate. Elevation of BNIP3/BNIP3L upon hypoxia triggers the displacement of Beclin from Bcl-2 and BCL-xL. This is achieved because Bcl-2 and BCL-xL preferentially bind on BNIP3/BNIP3L compared to Beclin. So, hypoxia-induced BNIP3/BNIP3L elevation disengages Beclin1. The unbound, free form of Beclin1 is active to induce autophagy while BNIP3 and BNIP3L are now “occupied” by BCL-xL/Bcl-2. Additionally, formation of the latter complexes inhibits cell death upon hypoxia (Bellot et al., 2009). The BH3 domains of these factors are critical for the formation of these complexes both in normoxia and hypoxia.

Additionally, it has been shown that BNIP3 physically interacts with Ras homolog enriched in brain (RHEB) and triggers a reduction of the RHEB GTP levels, thus inhibiting S6 kinase phosphorylation at Thr389 and ultimately mTOR (Li et al., 2007). This is another mechanism through which BNIP3 triggers general autophagy induction upon hypoxia. Further, it has been proposed that increased expression of BNIP3 and NIX in response to hypoxia causes mitochondrial depolarization and generalized mitochondrial dysfunction (Rikka et al., 2011). This, results in excessive ROS production which causes autophagy induction once again. These actions combined suggest that BNIP3-mediated elevation of general autophagy probably facilitates mitophagy induction as well. To this extent, a question arises relative to ROS homeostasis, BNIP3/NIX regulation, and HIF-1 activation. The notion that prevails up to now is that ROS is the main trigger for HIF-1-stabilization and nuclear localization. HIF-1 nuclear localization and activation triggers the expression of its target genes including BNIP3 and NIX. Albeit, the last example of BNIP3/NIX- mediated autophagy induction supports the idea that BNIP3/NIX elevation precedes ROS augmentation. An interesting question to consider, is whether the aforementioned mechanisms through which BNIP3 regulates autophagy upon hypoxia act in the same or in compensatory pathways.

Interestingly, BNIP3/NIX function as mitophagy receptors apart from their role in general autophagy. The function of both proteins in mitophagy is enhanced upon hypoxia/reoxygenetion. This coupling was initially shown in MEFs where BNIP3 was found to be both necessary and sufficient to trigger mitophagy upon hypoxia and equivalently reduce mitochondrial mass and overall functionality in terms of mitochondrial respiration (Zhang et al., 2008). BNIP3/NIX -induced mitophagy upon hypoxia additionally requires the homodimerization of BNIP3 and the activity of essential autophagy components such as Beclin-1and ATG5 (Hanna et al., 2012). Moreover, both BNIP3 and NIX contain a LIR motif, which is exposed in the cytoplasm, allowing for their physical interaction with LC3/GABARAP (Novak et al., 2010). Despite the fact that an integrated mechanistic insight relative to BNIP3/NIX-induced mitophagy upon hypoxia is still missing, several phosphorylation sites on BNIP3/NIX are decisive for the function of those receptors and for mitophagy induction. First, BNIP3L/NIX phosphorylation at Ser81 seems to be needed for the induction of mitophagy under ischemia-induced conditions although the responsible kinases still remain uncharacterized (Yuan et al., 2017). Second, two phosphorylation events, one at Ser17 and the other at Ser24 of BNIP3 strongly enhance its interaction with the Autophagy-related protein 8 (Atg8) members, LC3B and Golgi-associated ATPase Enhancer of 16 kDa (GATE-16), thus promoting mitophagy. Interestingly, BCL-xL triggers BNIP3-mediated mitophagy in a BH3-dependent manner. Further evidence leads to the conclusion that BNIP3-mediated mitophagy most likely acts as a protective mechanism controlling mitochondrial turnover and counteracting cytochrome c release (Zhu et al., 2013; Liu and Frazier, 2015).

Despite the fact that the pro-survival role of BNIP3 exerted through the control of mitophagy has been extensively tested under hypoxic conditions, still clear evidence regarding the regulation of this receptor upon hypoxic conditions is missing. Furthermore, enzymes that are expected to regulate both the protein levels and the receptor activity upon hypoxic versus normoxic conditions, such as kinases, phosphatases, and E3 ubiquitin ligases have not been identified yet. Interestingly, it has been shown in cardiomyocytes that BNIP3 induction triggers the translocation of Drp1, from the cytoplasm to mitochondria, resulting in mitochondrial fragmentation and subsequently, mitophagy induction (Lee et al., 2011). Importantly, Drp1 localization to mitochondria and mitochondrial fission seem to be a prerequisite for BNIP3-mediated mitophagy in cardiomyocytes. To date, however, strong evidence for direct coupling of Drp1 with BNIP3-mediated mitophagy upon hypoxia is still missing.

Furthermore, despite the fact that the involvement of PINK1/Parkin in hypoxia-induced mitophagy was initially excluded, latest evidence prompted researchers to revisit this theory. Specifically, it was reported that BNIP3 triggers both translocation of Pink1 to mitochondria and elevation of ubiquitination levels in cardiomyocytes (Lee et al., 2011). Moreover, it was recently shown in HEK293 cells that BNIP3 physically interacts with the full-length PINK1 on the OMM, despite the fact that it is not identified yet whether this interaction is direct or not. BNIP3-PINK1 interaction promotes PINK1 stabilization by blocking its proteasomal degradation. Stabilization of PINK1 on the OMM can then trigger Parkin and downstream processes including ubiquitination of OMM proteins which are targeted for degradation. Interestingly, PINK1 deletion did not completely abrogate mitophagy events, implying that BNIP3 can itself induce mitophagy by direct binding on LC3 and/or gamma-aminobutyric acid receptor-associated protein (GABARAP) in a PINK1- independent manner. It is interesting though, that while perturbation of BNIP3 did not have any effect on PINK1/Parkin-mediated mitophagy upon CCCP treatment it did affect hypoxia-induced mitophagy. In response to hypoxia, mitophagy in MEFs is induced through BNIP3-dependent accumulation of PINK1 on mitochondria. On the other hand, BNIP3 depleted cells did not exhibit neither PINK1 accumulation nor mitophagy. So, hypoxic induction of BNIP3 triggers the elevation of PINK1 protein levels and mitophagy (Zhang et al., 2016). This finding contradicts previous research showing that Pink1 deletion did not affect BNIP3-mediated mitophagy upon hypoxia. This discrepancy raises questions relative to whether Pink1 involvement in BNIP3 mitophagy is altered in a cell-type specific manner or whether it depends on the hypoxic conditions applied each time. Another node to the PINK1/Parkin participation in the hypoxic response is added by observations suggesting that Parkin can control HIF-1 and HIF-3 protein levels differentially in normoxia compared to hypoxia. As shown, loss of Parkin increases HIF-1 expression although it decreases HIF-3 in normoxia compared to the control. On the other hand, loss of Parkin upon hypoxia significantly reduces HIF-1 protein levels and also affects its subcellular localization (Maugeri et al., 2016). These data raise the possibility that a feedback loop that coordinates HIF-1, Pink1/Parkin levels and mitophagy exists. However, the possibility that Parkin obtains additional functions cannot be excluded.

**Figure 3:**
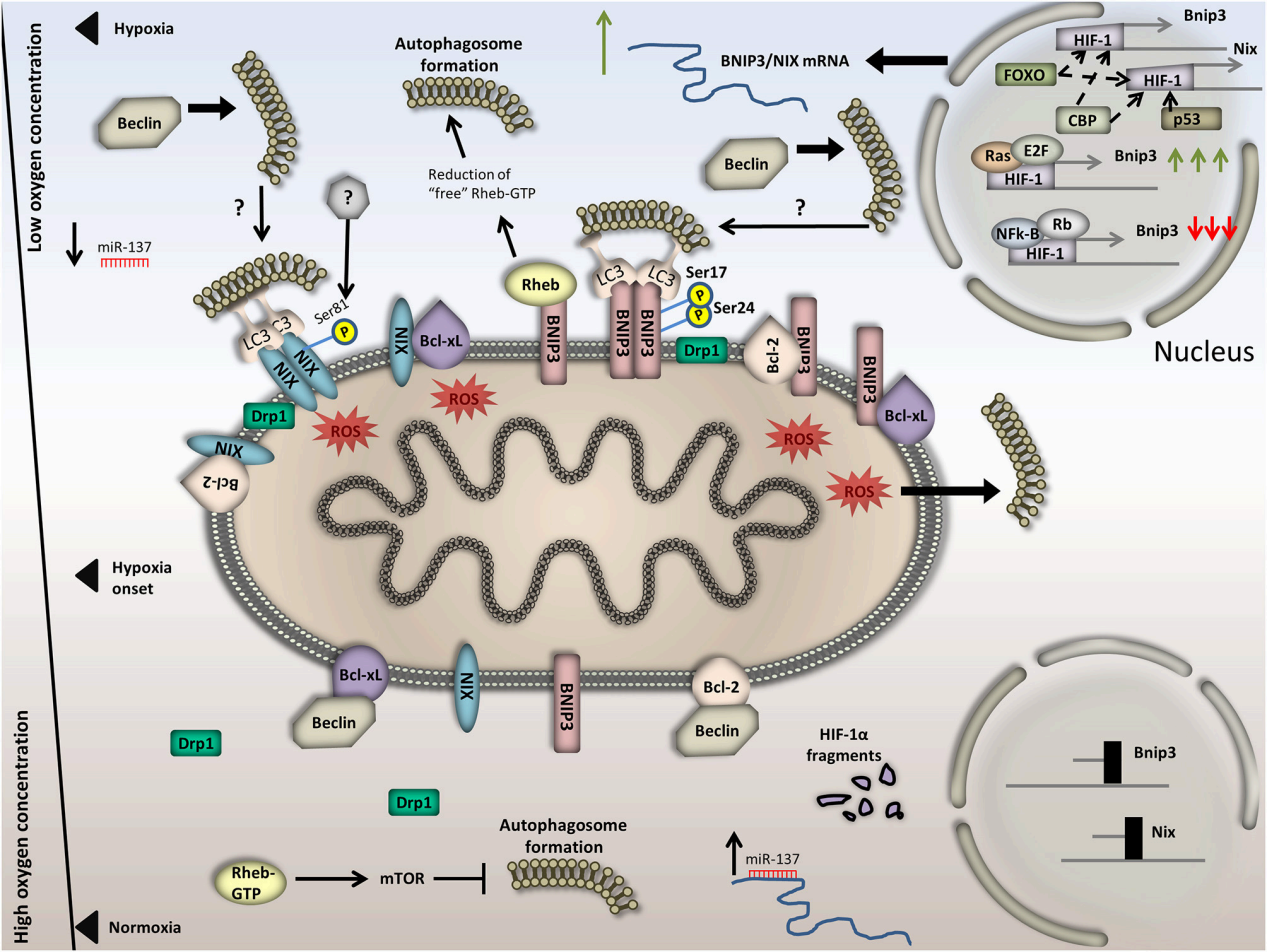
Regulation of BNIP3/NIX-mediated mitophagy in normoxia versus hypoxia. Under normoxia, BNIP3/NIX exhibit basal expression due to HIF-1 degradation in the cytoplasm and hence decreased transcriptional activity. Besides, the OMM localized BCL-xL and Bcl-2 tether Beclin and finally block autophagy induction. In addition, Rheb is free of BNIP3 binding, thus activates mTOR and blocks autophagy induction. Moreover, miR-137, which binds on the 3′ UTR of BNIP3/NIX and suppresses their expression, increases; and last, Drp1 dissipates in the cytoplasm. Upon hypoxia, on the other hand, BNIP3/NIX are highly expressed in a HIF-1-dependent manner. Increased BNIP3/NIX abundance on the OMM triggers Bcl-2/Beclin and BCL-xL /Beclin complex dissociation. Particularly, BNIP3 and NIX are bound on either BCL-xL or Bcl-2, rendering Beclin free to trigger autophagy induction. General autophagy induction could in turn trigger mitophagy. Additionally, BNIP3/NIX accumulation on the OMM triggers mitochondrial dysfunction and membrane depolarization. This leads to excessive ROS production, which can also activate general autophagy. Moreover, BNIP3 binds Rheb, thus diminishes the amount of “free”/cytoplasmic Rheb-GTP and inactivates mTOR, inducing again general autophagy. Concomitantly, BNIP3/NIX phosphorylation and dimerization triggers LC3 binding and finally mitophagy induction. It is also conceivable that Drp1 triggers mitochondrial fragmentation by translocating on the OMM. miR-137 levels drop. In the bottom half of the Figure, processes that take place under normoxia are illustrated and in the top half, processes under hypoxia. The axis in the left part of the Figure is representative of the available oxygen concentration.

At the post-transcriptional level, BNIP3L/NIX is regulated by miR-137, similarly to FUNDC1. miR-137 functions as a negative regulator that when overexpressed, decreases NIX protein levels and mitophagy as shown in HeLa, SKNSH, SY5Y, and HEK293 cells. This effect is very well correlated with hypoxia-induced mitophagy as hypoxia abrogates miR-137 expression (Li et al., 2014). The graphical representation of these mechanisms is shown in Figure 3. Evidence up to now suggests that BNIP3-induced mitophagy functions independently from FUNDC-1 mediated mitophagy upon hypoxia (Liu et al., 2012). To this extent, whether the BNIP3- versus BNIP3/PINK1- and FUNDC1-mediated mitophagy are induced upon different hypoxic conditions or in different cell types needs to be tested. For example, in UCB-hMSC cells all *PINK1, BNIP3* and *NIX* are transcriptionally upregulated in response to hypoxia, in contrast to *FUNDC1* which is downregulated (Lee et al., 2017). Also, it is not clear yet whether the PINK1/Parkin activation downstream of BNIP3 is a cellular response to enhanced mitophagy needs. In this respect, it is possible that additional to BNIP3/Nix-mediated mitophagy, activation of Pink1/Parkin-mitophagy serves as a mechanism to boost mitophagy events upon hypoxia. Verification of this hypothesis would highly increase our understanding of the mechanisms that regulate the mitochondrial pool in response to oxygen deficiency.

## Hypoxia-induced degradation of other organelles

The wide variety of metabolic alterations induced by hypoxia are expected to totally reorganize cellular function and affect several if not all cellular compartments in terms of abundance and/or function. Hence, additional targets other than mitochondria are expected to be regulated through selective autophagy upon hypoxia. Toward this direction, evidence exists that selective autophagy of the nucleus (nucleophagy), lipids (lipophagy), ribosomes (ribophagy), ER (ERphagy or reticulophagy), and/or peroxisomes (pexophagy) are activated upon hypoxic stimuli (Carloni et al., 2014; Chen K. et al., 2014; Rashid et al., 2015; Schönenberger et al., 2015; Li L. et al., 2016; Ma et al., 2018). The physiologic relevance and the exact mechanisms governing these selective autophagy types under hypoxia are not well understood yet. For this reason, we will discuss the most important findings on pexophagy and reticulophagy and provide possible future perspectives.

### Pexophagy

Peroxisomes are metabolically responsive and highly dynamic organelles in terms of size, number and function. Their key functions are oxygen-dependent and related to lipid synthesis, ROS metabolism and the degradation of both polyunsaturated fatty acids (PUFAs) and very long fatty acids (VLCFAs), among others (Berger et al., 2016). Peroxisomes produce H_2_O_2_ as a byproduct of their function which is either consumed in downstream reactions or released in tissues (Elsner et al., 2011). To this extent, cell adaptation to oxygen deficiency is expected to seriously readjust peroxisomal number and function. Upon hypoxia, peroxisomes are targeted for selective autophagy, named pexophagy. Through pexophagy induction, cells decrease peroxisomal number and downregulate the high-oxygen demanding processes which take place inside these organelles. This diminishes the cell demands for oxygen and renders them able to preserve their homeostasis even in conditions where oxygen is scarce.

Initial studies in the liver, where peroxisomes are mostly abundant showed that their number drops significantly in a HIF-2a/EPAS1-dependent manner. Despite the fact that this decrease was observed in a von Hippel–Lindau (Vhl) mutant background where HIFs are constitutive active, HIF-1 did not exhibit any involvement in the regulation of peroxisomal number. Since a receptor for HIF-2α -dependent pexophagy under the conditions tested was not identified, it was speculated that the general autophagy receptors Neighbor of BRCA1 gene 1 protein (NBR1) and p62 mediate the effect (Deosaran et al., 2013). Indeed, Nbr1 and p62 are localized on peroxisomes upon HIF-2a stabilization, although Nbr1 was also found there when oxygen is abundant (Walter et al., 2014). Supportive evidence showed that HIF-2α overexpression triggered the concomitant drop of both the peroxisomal number and NBR1 levels. Moreover, both components recognize ubiquitinated proteins on the peroxisomal outer membrane and bind on them. Receptor binding of ubiquitinated substrates subsequently triggers autophagosome formation and engulfment of the organelle. Interestingly, both NBR1 and p62 are degraded in a ROS-dependent manner, although a correlation between ROS, NBR1/p62 and hypoxia has not been established yet (Ishaq et al., 2014).

The field of hypoxia-induced pexophagy is an expanding field in which current understanding is limited. Detailed mechanistic insight would offer better understanding of the hypoxia response mechanisms owing to the fact that peroxisomal function is a determinant of cellular homeostasis. Toward this direction, the identification of specific peroxisomal proteins that function as pexophagy receptors is important. To date, the only specific pexophagy receptors that have been identified are Atg30 and Atg36 in yeast, both of which do not have mammalian orthologs (Farré et al., 2008; Motley et al., 2012). Furthermore, the mechanism by which NBR1 and p62 regulate hypoxia-induced pexophagy is not understood. Moreover, direct evidence for NRB1 and p62 binding on specific ubiquitinated targets does not exist since neither such substrates nor the responsible E3 ubiquitin ligases have been identified. This raises the possibility that these receptors could regulate pexophagy even in a ubiquitin-independent manner.

Our knowledge relative to peroxisomal proteins that participate in pexophagy is still very limited. Only Peroxisomal E3 ubiquitin ligase peroxin 2 (PEX2), which targets PEX5 and 70-kDa peroxisomal membrane protein (PMP70), is a verified peroxisomal component that functions as a pexophagy receptor under starvation conditions. Whether PEX2 mediates hypoxia-induced pexophagy has not been studied yet (Sargent et al., 2016). Interestingly, hypoxia induction downregulates Pex5 in glioblastoma cancer cells but it is not known yet whether this effect is PEX2-dependent (Huang et al., 2012). Moreover, in response to excessive ROS, PEX5 is phosphorylated at S141 by Ataxia-telangiectasia mutated (ATM) kinase, which translocates from the nucleus to peroxisomes to bind on PEX5. This phosphorylation event subsequently triggers PEX5 monoubiquitination at K209 and activates p62-mediated pexophagy although NBR1 contribution was not tested (Zhang et al., 2015).

ATM signaling also triggers autophagy in a ROS-dependent manner, through both ULK1 activation and mTORC1 inhibition. We speculate that this mechanism could also apply for hypoxic conditions where ROS, ULK1, and mTOR obtain a primary role. Another issue that should be addressed by future studies is the mechanism by which HIF-2α triggers pexophagy. Moreover, taking into account that hypoxic responses depend on ROS and that ROS is also the main stimulus of pexophagy makes the possibility that ROS is the triggering mechanism of pexophagy upon hypoxia very appealing. Notably, the need of ROS for pexophagy induction is further strengthened, as it was shown recently that both genetic and pharmacologic inhibition of catalase, a peroxisomal protein that eliminates ROS and specifically H_2_O_2_ generated by peroxisomes, triggers pexophagy, and increases ROS levels in HepG2 cells. In fact, initiation of pexophagy upon catalase depletion is ROS- dependent as concomitant treatment with the antioxidant N-acetylcysteine (NAC) abolished pexophagy (Jo et al., 2015; Lee et al., 2018). The factors mentioned in this section and relative information are summarized in Table 1.

**Table 1:** Components that are implicated in pexophagy and their association with hypoxia.

**Gene name**	**Function**	**Additional comments**	**Direct/Indirect association with hypoxia**
NBR1	Receptor	Recognizes ubiquitinated proteins	Enriched on peroxisomes upon HIF inducing conditions ROS dependent degradation
p62	Receptor	Recognizes ubiquitinated proteins	Enriched on peroxisomes upon HIF inducing conditions ROS dependent degradation
PEX2	E3 ubiquitin ligase Receptor	Binds on PEX5 and PMP70	Not studied
PEX5	Import receptor	Shuttles between the peroxisomal outer membrane and the cytoplasm Mono- and poly-ubiquitinated	Downregulated upon hypoxia Phosphorylated in a ROS-dependent manner and triggers pexophagy
ATM	Kinase	Phosphotylates Pex5	ROS dependent

### ERphagy

Elevation of UPR has been linked to the downstream induction of selective autophagy of the ER, named ERphagy/reticulophagy (Li L. et al., 2016). It is now well established that ER stress is highly induced upon hypoxia, but evidence showing a direct link with ERphagy or reticulophagy is still scarce. Early studies in human cells have revealed the existence of four proteins that specifically function as ERphagy receptors: cell-cycle progression gene 1 (CCPG1), JK-1(FAM134B), SEC62, and Reticulon-3 (RTN3). Interestingly, BNIP3 that was shown previously to participate in mitophagy induction upon hypoxia seems to play a role in ERphagy regulation as well; but the mechanism of action for all the above receptors is still unknown. Additionally, evidence from studies performed in yeast cells, suggests that autophagy of the ER relies on a novel mechanism, independent of the core autophagic machinery (Schuck et al., 2014). Current understanding shows that both RTN3 and FAM134B participate in the starvation-induced ERphagy. Furthermore, SEC62 participates in a specific type of ERphagy that is activated upon acute ER stress in order to alleviate disturbances and re-establish normal homeostasis. Last, CCPG1 is also a stress-induced receptor, but due to the fact that it was only recently identified, still little is known about its mechanism of action (Khaminets et al., 2015; Fumagalli et al., 2016; Grumati et al., 2017; Smith et al., 2018). Notably, FAM134B was found to be among a subset of genes that are upregulated in chronic myeloid leukemia (CML) cells and is correlated with pro-survival phenotypes and poor prognosis. Its upregulation is most probably HIF-1- dependent, like the upregulation of other genes in the same functional subgroup (Ng et al., 2014).

Accumulating evidence showing that Sec62 is highly elevated in the tumor microenvironment point toward a HIF-1-dependent regulation of Sec62 upon hypoxia induction (Linxweiler et al., 2012; Wemmert et al., 2016). Interestingly, Sec62 mediates the translocation of newly synthesized proteins into the ER. This function is achieved through a Sec61-Sec62-Sec63 complex formation on the ER membrane. Especially, the association of Sec62-Sec63 is enhanced by three phosphorylation events on Sec63 at serine residues 574, 576, and 748 by the CK2 kinase (Ampofo et al., 2013). Taking into account that CK2 kinase is responsive to alterations in the oxygen levels, as was observed in the FUNDC1 model upon hypoxia, one would speculate that CK2 could regulate ERphagy in response to hypoxia through a similar mechanism (Mottet et al., 2005). Interestingly, CK2 levels are elevated upon hypoxia and CK2 itself phosphorylates and enhances HIF-1 activity (Mottet et al., 2005; Hubert et al., 2006; Sermeus and Michiels, 2011). On the other hand, the ERphagy receptor RTN3 was found to be downregulated in response to hypoxia in human monocyte-derived macrophages *in vitro* (Fang et al., 2009). In addition, RTN3 protein levels where decreased in fetal heart tissue of sheep exposed to hypoxia, implying the existence of a global mechanism that despite triggering the rest of the ERphagy receptors, downregulates RTN3 (Li et al., 2018). To which extent does RTN3 downregulation affect the overall ERphagy levels upon hypoxia, which is the physiologic relevance of this downregulation and whether the effect is cell-type specific or not needs to be studied in the future. Interestingly BNIP3, which functions as a mitophagy receptor upon hypoxia and is a HIF-1 target gene, has been reported to also function as an ERphagy receptor in HeLa cells (Hanna et al., 2012). Altogether, evidence points toward a physiologic relevance between ERphagy and hypoxia (Table 2). We suggest that ERphagy plays a significant role under hypoxic conditions, supported by the emerging roles of ERphagy receptors in pathological conditions such as cancer.

**Table 2:** Components that are implicated in ERphagy and their association with hypoxia.

**Gene Name**	**Function**	**Additional comments**	**Association with hypoxia**
CCPG1	Receptor	Stress-induced, not well studied yet	Not studied
FAM134B	Receptor	Starvation-induced ERphagy	HIF-1 dependent Increased in CML
SEC62	Receptor	ER stress induced ERphagy Sec62 mediates the translocation of newly synthesized proteins into the ER	Highly increased in tumor micro-environment
RTN3	Receptor	Starvation-induced ERphagy	Downregulated upon hypoxia in human monocyte-derived macrophages and sheep fetal heart tissue
BNIP3	Receptor	It also regulates mitophagy upon hypoxia	HIF-1 target gene
CK2	Kinase	Phosphorylates Sec63	Oxygen-dependent (altered)functionality Promotes HIF-1 activity

## Hypoxia and cancer

Cancer cells have the ability to rapidly proliferate and divide giving rise to various types of tumors depending on the tissue of origin. Tumor microenvironment within a solid tumor is characterized by extreme heterogeneities due to the distance a cancer cell obtains from blood vessels. Blood vessels are mostly evident in the periphery of the tumor and function as suppliers of oxygen and other nutritional material to neighboring cells, thereby promoting their proliferation. In contrast, cells located in the more central areas of the solid tumor are often challenged with low oxygen levels. Oxygen scarcity activates HIFs; HIF activation totally alters the metabolic profile of tumor cells by lowering oxidative phosphorylation and promoting glycolysis. Apart from the altered metabolism, activation of additional HIF targets can in parallel promote both vasculogenesis and angiogenesis (Krock et al., 2011). Thus, cells are locally supplied with oxygen and nutrients, boosting their previously stalled proliferation. This is a never-ending phenomenon, as proliferation would again raise oxygen needs, re-creating a hypoxic microenvironment in a constrained part of the tumor due to the local, abrupt expansion of cells. This re-activates HIFs further promoting tumor expansion, aggressiveness, metastasis, and drug resistance. Taking into account the importance of HIF-1 responses in tumor progression and patient prognosis, it is crucial to gain deep understanding of the mechanisms that are activated downstream of HIFs.

## Selective autophagy components in cancer

The cell adapts to hypoxic stimuli through the activation of delicate mechanisms which converge on autophagy for recycling of unwanted components or/and organelles, contributing to the preservation of homeostasis. Dysfunction of such mechanisms is coupled with the onset of severe human pathologies such as cancer. In the following section, we will outline recent findings indicating a tight coupling of the aforementioned selective autophagy components to cancer metabolism, emphasizing on studies in mammalian cells.

### Mitophagy components in cancer

Mitochondrial function is of exceptional importance for cellular and organismal health. Dysregulated mitochondrial homeostasis triggers mitophagy, a process needed to clear damaged mitochondria and prevent their accumulation. Mitophagy impairment leads to accumulation of toxic mitochondrial metabolism byproducts such as ROS that further induce DNA damage and lead to tumorigenesis. Interestingly, components that mediate mitophagy have upcoming roles in several types of cancer. For example, BNIP3 and NIX are highly expressed in breast, macrophage, endothelial and epithelial cancer cells compared to healthy cells from the same patient upon hypoxia induction (Sowter et al., 2001). Furthermore, BNIP3 is highly expressed in lung cancers and follicular lymphomas. On the other hand, BNIP3 is not expressed in other types of cancers such as the pancreatic, colorectal and gastric cancer even under hypoxic conditions. In most pancreatic tumors BNIP3 is methylated. Methylation prevents HIF-1 transcription factor binding, thus inactivating BNIP3. This phenomenon was also observed in many cases of primary colorectal, acute lymphotic, gastric cancer, and multiple myelomas (Li Y. et al., 2017).

BNIP3 loss in pancreatic cancer has been associated with decreased apoptosis in tumor cells, metastatic phenotypes and poor prognosis, rendering BNIP3 a possible anti-tumor gene for this kind of malignancy (Okami et al., 2004; Chourasia et al., 2016; Li Y. et al., 2017). In colorectal cancer, BNIP3 silencing was correlated with increased cell growth and resistance to chemotherapy. In this case, BNIP3 downregulation was associated with aberrant methylation mediated by DNA-methyltransferase 3 beta (DNMT3B) and DNA-methyltransferase 1 (DNMT1) (He et al., 2017). Most possibly, the subcellular localization of BNIP3 is also a measure of functionality. For example, in glioblastoma tumor cells, despite the fact that BNIP3 levels were elevated in the hypoxic areas of the tumors, its localization was not mitochondrial or cytoplasmic as expected, but nuclear. It is not clear whether BNIP3 also has an additional, unknown function in the nucleus, but current evidence suggests that its nuclear localization is a sign of dormancy (Burton et al., 2006). This is in line with observations that BNIP3 is found mainly in the cytoplasm in invasive human breast cancer cells, while in healthy cells BNIP3 is predominantly localized to the nucleus. The physiologic relevance of these observations is not understood yet, although it could reflect the activity status of BNIP3. As previously mentioned, the subcellular localization of BNIP3 protein is altered in invasive human breast cell carcinomas compared to healthy cells and this was oppositely correlated with HIF-1 expression, tumor progression, and good prognosis (Koop et al., 2009). On the other hand, BNIP3 was mainly localized to the nucleus and less in the cytoplasm of laryngeal squamous cell carcinoma (SCC) tumor cells (Jin et al., 2012).

Moreover, BNIP3 is proportionally increased both at the protein and mRNA level by the oncogene Ras. Even in the absence of hypoxic conditions, Ras activation or overexpression could increase BNIP3 levels. This phenomenon was evident in breast, lung, prostate cancer and kidney adenocarcinoma as well as in leukemia (Kalas et al., 2011). On top of that, BNIP3 transcriptional activation by HIF-1, FOXO3A, and E2F is highly induced when Ras is activated (Kalas et al., 2011). Furthermore, microarray analysis performed in patients with renal cell carcinoma (RCC) indicated that increased cytoplasmic levels of BNIP3 correlated with metastasis and poor prognosis implicating that BNIP3 acts as a pro-survival factor and its levels could be used as a prognostic marker for this type of cancer (Macher-Goeppinger et al., 2017). Similar experiments performed in melanoma cell lines under hypoxia revealed a significant increase in BNIP3; an effect that is correlated with poor prognosis and resistance to pembrolizumab (anti-PD1) immunotherapy (Buart et al., 2017). The effect of BNIP3 does not seem to equally apply in every type of breast tumor. In contrast to other studies, in breast cancer cells, BNIP3 deletion promotes metastasis and is linked with poor prognosis in human triple-negative breast cancer (TNBC). In addition, downregulation of the tumor suppressor retinoblastoma protein highly induces BNIP3 expression under hypoxic conditions (Tracy et al., 2007). On the other hand, the tumor suppressor p53 can directly bind to the BNIP3 promoter and block its expression both under normoxic and hypoxic conditions, thereby inhibiting hypoxia-induced BNIP3-autophagy induction (Feng et al., 2011). Since BNIP3 and NIX are both involved in mitophagy and apoptotic cell death, it is possible that their role may vary depending on the tumor type. For example, in one cancer type they may exert their role through mitophagy, in another through apoptosis and in other cases the balance between mitophagy and apoptosis may determine tumor progression. Future studies are expected to shed light in such speculations.

As far as FUNDC1 is concerned, it was lately shown that cervical cancer cells obtained from early-stage patient tissues had significantly higher levels of the protein compared to adjacent normal cells. Interestingly, this high FUNDC1 expression was negatively correlated with tumor progression and patient prognosis whereas reduction of FUNDC1 levels halted cancer cell proliferation and in parallel induced apoptosis as well as sensitivity to both cisplatin and ionizing irradiation (Hou et al., 2017). Moreover, studies on the PGAM5/FUNDC1/BCL-xL/DRP1 axis described previously in non-small cell lung cancer points toward the direction that targeting mitophagy through FUNDC1 in combination with X-ray irradiation could improve treatment of this type of human cancer (Dong et al., 2017). Additional evidence correlates FUNDC1 and PGAM5 expression with NSCLC and macrophages. Toward this direction, both FUNDC1 and PGAM5 are only expressed in NSCLC epithelial cells and the adjacent macrophages which through yet unknown mechanisms sent signals to neighboring cancer cells, thus determining their fate (Ng Kee Kwong et al., 2017).

Additional factors that regulate hypoxia-induced mitophagy play important roles in cancer cell homeostasis and tumor progression such as the aforementioned kinases Src and CK2. Both kinases retain oncogenic roles and are important players in several types of tumors as reviewed elsewhere, although whether their effect on tumorigenesis is mediated through their role in mitophagy or through other functions has not been well studied (Kim et al., 2009; Trembley et al., 2009, 2010; Zhang and Yu, 2012; Chen et al., 2018). Moreover, CNX, another component implicated in hypoxia-induced mitophagy, is highly increased in cancer cells. This characteristic could render CNX a valuable prognosis marker (Lakkaraju and van der Goot, 2013; Kobayashi et al., 2015; Ryan et al., 2016; Ma et al., 2017). On the other hand, miR-137 acts as a tumor suppressor as evidenced in various cancer cell types (Neault et al., 2016; Chen T. et al., 2017; Ding F. et al., 2018). Finally, the role of the PINK1/Parkin pathway in cancer onset and progression has already been extensively reviewed (Lu et al., 2013; Matsuda et al., 2015; Eid and Kondo, 2017; Palikaras et al., 2017). Interestingly though, it was found that ARIH1 substitutes Parkin in the PINK1/Parkin-mediated mitophagy that takes place specifically in cancer cells. Besides, it is proposed that ARIH1 can be used as a prognostic marker for chemotherapy, as already tested in lung adenocarcinoma patients (Villa et al., 2017).

### Pexophagy components in cancer

Lately, pexophagy has also been implicated in tumorigenesis and tumor progression. Toward this direction, it is not only the fact that pexophagy rates are highly induced in a HIF-1- and oxygen-dependent manner but also that specific pexophagy components have been implicated in the regulation of tumor homeostasis. For example, Nbr1 and p62 which both function as pexophagy receptors, play a role in cancer homeostasis. Specifically, Nbr1 is expressed in the cytoplasm of low-grade non-musical-invasive bladder cancer cells and is correlated with poor prognosis (Chi et al., 2017). On the other hand, it was recently identified that Nbr1 expression is downregulated in clear cell renal cell carcinoma (ccRCC), phenomenon that is correlated with poor patient prognosis and resistance to sunitinib treatment. As a result, Nbr1 could possibly be used as a prognostic marker for both metastasis and chemoresistance in patients with this type of malignancy (Ruan et al., 2017).

Nbr1 also promotes cell migration and regulates focal adhesion in a breast cancer cell line (Kenific et al., 2016). Furthermore, Nbr1 transcript levels are highly decreased in mammary cancer cell lines compared to their healthy counterparts (Dimitrov et al., 2001). Whether this affects breast cancer progression and prognosis or whether pexophagy is affected and plays a crucial role is expected to be answered in the future. Additional involvement of Nbr1 and p62 to cancer metabolism is indicated through their responsiveness to several compounds with anticancer properties. For example, Gambogic acid (GA), an anti-tumor drug and ROS inducer, cleaves and inactivates both p62 and Nbr1, among others, through ROS-mediated caspase activation (Ishaq et al., 2014). Furthermore, testing for possible anti-tumor effects of copper (I) nicotinate complex (CNC) on squamous cell cancer revealed that the drug could decrease general autophagy levels and elevate Nbr1 expression through yet unknown mechanisms (Abdel-Mohsen et al., 2017).

Except for the aforementioned factors, PEX2 expression is also highly increased in hepatocellular carcinoma (HCC) cells compared to healthy cells. It was shown that increased PEX2 expression was correlated with enhanced tumor growth whereas its depletion leads to increased ROS production, ER stress and autophagy induction. Similar effects were observed for PEX10 and PEX12 (Cai et al., 2018). These findings indicate that liver cancer may behave differentially than other cancer types as in this case ROS and autophagy induction lead to cell death and not to tumor progression. It would be interesting to study whether the PEX2- dependent liver cancer progression is HIF-1-dependent or not.

Furthermore, Pex5 and PMP70 are also implicated in cancer progression. For example, Pex5 mRNA levels were significantly increased in colon carcinoma cells, while they were decreased in C6 glioma cells exposed to hypoxia (Lauer et al., 1999; Huang et al., 2012). Furthermore, the mRNA levels of PMP70 were unchanged and the protein levels of PMP70 decreased in colon carcinoma cells (Lauer et al., 1999). Following, overexpression of the tumor suppressor H-rev107 triggered the absence of PMP70 from peroxisomes in human embryonic kidney cells 293 (HEK293 cells) (Uyama et al., 2012). Also, PEX3 downregulation reduced the resistance of lymphoma cells to Vorinostat (Vor) by triggering apoptosis (Dahabieh et al., 2017). Furthermore, mutations on the ATM kinase gene are highly oncogenic, predictive of poor prognosis conferring resistance toward therapeutic approaches in various types of cancer such as colorectal, breast, lung and hematopoietic (Squatrito et al., 2010; Feng et al., 2015; Stagni et al., 2015; Weber and Ryan, 2015; Antonelli et al., 2017). Despite the fact that ATM is characterized as a tumor suppressor gene, its activity is not uniform in every type of cancer. Importantly, it was shown that ATM depletion inhibited tumor progression and metastasis in colon cancer cells (Liu et al., 2017).

### ERphagy components in cancer

ER is one of the most important organelles for cellular homeostasis. Its importance is underscored by the fact that intricate stress response mechanisms have been developed and are activated soon after hypoxia onset. Moreover, most of the proteins implicated in ERphagy are associated with cancer. For example, the recently identified ERphagy receptor CCPG1 has been linked to prostate cancer and in fact was proposed as a predictive biomarker for this type of cancer (Rizzardi et al., 2014). Furthermore, CCPG1 was shown to physically interact with both FIP200 and ATG8 in a lung cancer cell line, thus possibly directly affecting autophagy initiation (Smith et al., 2018). Moreover, CCPG1 was found to be downregulated in colon cancer (Gavert et al., 2013). Finally, downregulation of CCPG1 in retina retinoblastoma cells is correlated with cell proliferation and decreased apoptotic cell death, an effect that is mediated by miR-498 (Yang et al., 2018). Interestingly, miR-498 is downregulated in several types of cancers such as ovarian, non-small cell lung and colon cancers, an effect that is correlated with poor prognosis (Gopalan et al., 2015; Liu et al., 2015; Wang et al., 2015). Whether miR-498 downregulation in the aforementioned types of cancers affects tumor progression through CCPG1 remains to be identified.

Moreover, evidence linking FAM134B, another ERphagy receptor, with cancer recently came to light. Specifically, it was found that decreased FAM134B expression in colorectal adenocarcinomas is coupled with enhanced tumor aggressiveness, poor prognosis, and tumor re-occurrence as well as metastasis. Deeper analysis showed that in this type of cancer cells, FAM134B was inactivated through promoter methylation and interestingly this effect was found to be tumor stage—specific, i.e., late-stage cancer cells exhibited increased FAM134B promoter methylation in comparison to earlier ones (Islam et al., 2017, 2018). Moreover, in colon cancer cells, an alternative FAM134B inhibition pathway through miR-186-5p, has been revealed rendering FAM134B a tumor suppressor, at least for this type of tumor (Kasem et al., 2014; Islam et al., 2017). Additionally, FAM13B is mutated in about half of the colorectal cancer samples tested compared to their healthy counterparts. Different types of mutations were identified ranging from single-nucleotide substitutions to insertions and deletions, among others (Kasem et al., 2014; Islam et al., 2017). Mutations on FAM134B have been identified in other types of cancers as well, such as in oesophageal squamous cell carcinoma (Haque et al., 2016). The localization of FAM134B in colon cancer cells was both cytoplasmic and nuclear with the higher proportion found in the cytoplasm (Islam et al., 2017). Interestingly, FAM134B is a predicted target of an additional miR, namely, miR-4284. This miR is downregulated upon hypoxic conditions, in irradiation-resistant cells and in prostate cancer AMC-22Rv1 cells (McDermott et al., 2017). FAM134B obtains an oncogenic role in chronic myeloid leukemia (CML) cells under hypoxia. Specifically, FAM134B is upregulated in CML promoting cancer cell survival and drug resistance, ultimately associated with poor patient prognosis (Ng et al., 2014). These findings indicate the complex regulation imposed on FAM134B among the different types of cancers. Deeper understanding of the regulatory mechanisms would prove crucial for targeted and successful therapeutic interventions.

Sec62 which also plays a significant role in ERphagy has been correlated with various types of tumors. Sec62 is highly elevated both at the mRNA and protein levels in prostate cancers and is positively correlated with decreased apoptosis in thapsigargin-treated cells, whereas, downregulation of Sec62 makes cells more responsive to this type of therapy (Jung et al., 2006; Greiner et al., 2011). Also, Sec62 is upregulated in other types of tumors, such as the thyroid and non-small cell lung tumor. In all three types of tumors, blockage of Sec62 expression is very well correlated with loss of cell differentiation capacity, tumor invasiveness and metastasis, although cell viability was not significantly affected (Greiner et al., 2011; Körbel et al., 2018). Furthermore, Sec62 was also significantly elevated in more than 80% of thyroid and cervical cancers. In these types of tumors also, ER stress resistance and metastatic capacity were dependent on Sec62 increased protein levels (Linxweiler et al., 2012, 2016). Importantly, increased Sec62 protein levels are evident in post-surgical patients with HBV-related hepatocellular carcinoma recurrence. This finding implies not only that Sec62 could be used as a prognostic marker but also as a new therapeutic target for HCC recurrence (Weng et al., 2012). Additionally, Sec62 overexpression has been detected in head and neck squamous cell carcinomas. In these types of tumors, again, Sec62 overexpression is linked with lymphatic metastasis and poor patient prognosis (Wemmert et al., 2016; Bochen et al., 2017). The exact mechanism of Sec62 tumorigenic activity is not yet understood despite the fact that its role in various types of tumors render it an oncogene.

Finally, RTN3, another ERphagy receptor was found to be downregulated upon hypoxia, as previously mentioned. Despite the fact that information relative to its role in cancer is still limited, it was first shown that RTN3 overexpression triggers tumor necrosis factor-related apoptosis-inducing ligand (TRAIL)-, tumor necrosis factor (TNF)-α and Fas-dependent apoptosis. Interestingly, TRAIL selectively induces apoptosis of renal cancer cells without affecting the viability of healthy cells (Lee et al., 2009). These results imply that RTN3 could be used as a therapeutic target at least in this type of human cancer. Moreover, studies in HeLa cells revealed that RTN3 physically associates with Ras on the endoplasmic reticulum and proposed that RTN3, at least in the model tested, could regulate Ras localization and functionality. Specifically, it is speculated that RTN3 “traps” Ras on the ER, rendering it inactive, by disrupting its redistribution on the plasma membrane (Su et al., 2007). Next, RTN3 was recently identified as a novel prognostic marker for HCC together with UPB1 and SOCS2. RTN3 is positively correlated with HCC and its levels were significantly increased in tumor tissues compared to healthy ones. Additional studies are needed to verify the role of RTN3 in HCC and its mechanism of function (Li B. et al., 2017). Moreover, studies performed in cancer tissues from patients point toward an oncogenic role of RTN3, as it was shown that increased RTN3 levels are observed in astrocytoma whereas no expression was observed in healthy glial cells (Huang et al., 2004). Furthermore, RTN3 was one of the top three upregulated genes in chemotherapy-sensitive epithelial ovarian cancer samples pointing toward an anti-tumor role under these conditions (Zhang and Luo, 2016). These findings highlight the need for tight regulation of selective autophagy components within the tumor microenvironment. The expression changes of selective autophagy components observed in cancer versus healthy cells are summarized in Table 3.

**Table 3:** Expression patterns of selective autophagy components linked to hypoxia in various human cancers.

**Gene name**	**Expression pattern per cancer type**			
	**Increased**	**Decreased**	**Mutation**	**Not expressed**
BNIP3	Breast cancer, Macrophage cancer, Endothelial cancer, Epithelial cancer, Lung cancer, Follicular lymphomas, Glioblastoma, Prostate cancer, Kidney adenocarcinoma, Leukemia, Renal cell carcinoma, Melanoma			Pancreatic cancer, Colorectal cancer, Gastric cancer, Acute lymphoma, Myelomas
NIX	Breast cancer, Macrophage cancer, Endothelial cancer, Epithelial cancer			
FUNDC1	Cervical cancer			
Nbr1	Cervical cancer, Bladder cancer, Clear cell renal cell carcinoma	Breast cancer		
ATM			Breast cancer, Lung cancer, Colorectal cancer, Hematopoietic cancer	
PEX2	Hepatocellular carcinoma			
PEX5	Colorectal cancer			
CCPG1	Prostate cancer	Colorectal, Retina retinoblastoma		
FAM134B	Chronic myeloid leukemia	Colorectal cancer	Oesophageal squamous cell carcinoma	
Sec62	Prostate cancer, Cervical cancer, Thyroid cancer, Non-small cell lung cancer, Hepatocellular carcinoma, Head squamous cell carcinoma, Neck squamous cell carcinoma			
RTN3	Hepatocellular carcinoma, astrocytoma			

## Concluding remarks

Cells, tissues, and whole organisms may be physiologically exposed to hypoxia, as for instance occurs during embryonic development or when exposed to high altitudes. On the other hand, hypoxia is a common feature of several human pathologies such as ischemia and cancer and importantly stands as the causative link in their onset. Cells respond to hypoxia by adapting their metabolism and function through a number of hypoxia-associated pathways comprising HIFs, mTOR, UPR, and autophagy. HIFs activate several stress response mechanisms, most of which converge on autophagy, to restore homeostasis and ensure cell survival. Importantly, the fact that solid tumors are characterized by hypoxic microenvironment and exhibit HIF activation renders the comprehensive delineation of autophagy pathways necessary. Since autophagy functions as a pro-survival mechanism, its targeted downregulation is a common strategy applied for eliminating cancer cells or making them sensitive to chemotherapy. On the other hand, recent data indicate that this is not always the case. More specifically, it seems that at the stage before the proliferating cells become malignant, i.e., in healthy cells, autophagy induction has a protective, tumor-suppressive role whereas in advanced cancers its role can be both tumorigenic and tumor suppressive. Toward this direction, loss of Beclin1, Atg5, and/or Atg7 has been associated with the onset of several types of tumors. On the other hand, the means by which autophagy can become tumorigenic when activated in the tumor microenvironment is by promoting tumor cell survival and proliferation. At this point, blocking autophagy would be appealing, but concerns toward this direction have arisen. These are based on studies that highlight a possible intervention with anti-tumor inflammatory responses that would in the end convert such handlings from tumor suppressive to tumorigenic (Townsend et al., 2012; Rao et al., 2014).

It is becoming apparent that autophagy inhibition even in the same cell population can differentially impact cancer cell viability. Furthermore, autophagy inhibition at different stages of the tumorigenic process can erratically impact cell viability. All these issues raise the complexity of each tumor entity and render therapeutic strategies in many cases unpredictable. Intervention strategies that globally target the general autophagic machinery or mTOR are proven insufficient and risky, provoking severe side effects for the patient. It is possible that the best strategy for tackling tumor progression would be by regulating specific types of selective autophagy and not general autophagy components that would uniformly affect all types of selective autophagy. Moreover, it becomes apparent that altering a specific type of selective autophagy differentially impacts tumor progression. The same manipulation can either be tumor suppressive or tumorigenic and this is mainly dependent on the tumor stage and cell type. In this respect, we suggest that targeting selective autophagy components instead of general autophagy would be the best approach toward cancer treatment. Such therapies require the development of the appropriate drugs that most possibly would be best combined with chemotherapy or radiotherapy. Ideally, the effectiveness of such therapeutic approaches is expected to be significantly improved if seen in the context of personalized medicine.

## Author contributions

ID and IG wrote the manuscript. NT organized and edited the manuscript.

### Conflict of interest statement

The authors declare that the research was conducted in the absence of any commercial or financial relationships that could be construed as a potential conflict of interest.
